# The role of liquid biopsy in the management of concurrent Hodgkin lymphoma and ovarian carcinoma treated with nivolumab

**DOI:** 10.1007/s12672-025-03792-6

**Published:** 2025-11-07

**Authors:** Veronika Hanáčková, Jan Grohmann, Helena Urbánková, Hana Študentová, Eva Buriánková, Lenka Henzlová, Tomáš Papajík, Veronika Bachanová, Vít Procházka

**Affiliations:** 1 Department of Haemato-oncology, University Hospital Olomouc and Faculty of Medicine and Dentistry, Palacký University Olomouc, Olomouc, Czech Republic; 2 Department of Oncology, University Hospital Olomouc and Faculty of Medicine and Dentistry, Palacký University Olomouc, Olomouc, Czech Republic; 3 Department of Nuclear Medicine, University Hospital Olomouc and Faculty of Medicine and Dentistry, Palacký University Olomouc, Olomouc, Czech Republic; 4 Division of Hematology, Oncology and Transplantation, University of Minnesota, Minneapolis, MN USA

**Keywords:** Hodgkin lymphoma, Ovarian carcinoma, Circulating tumor DNA, Immune checkpoint inhibitors, Minimal residual disease

## Abstract

The simultaneous occurrence of Hodgkin lymphoma (HL) and ovarian carcinoma (OC) is rare and presents unique challenges in diagnosis and treatment. Circulating tumour DNA (ctDNA) offers a minimally invasive method for detecting minimal residual disease in both malignancies. We present the case of a 42-year-old woman with relapsed HL and advanced high-grade serous OC, treated with nivolumab, a PD-L1 inhibitor. After surgery and chemotherapy for OC, she received salvage therapy for cHL, including autologous stem cell transplantation. During the therapy, the patient was monitored using PET/CT and ctDNA analysis. CtDNA analysis detected a Hodgkin-driven compound mutation in the STAT6 gene (N417Y/N421S) allowing early relapse detection and treatment adjustments, detected progression of the disease led to nivolumab initiation. The mutation was used to monitor minimal residual disease (MRD). For ovarian carcinoma, presence of the BRAF V600E mutation as the marker was detected from archival paraffin-embedded ovarian tissue collected at the time of diagnosis, during ctDNA monitoring, BRAF V600E associated with OC remained undetectable, aligning with remission. This case highlights the potential of ctDNA to improve monitoring of concurrent malignancies, especially during immunotherapy, where PET/CT may lead to false-positive results. It is the first reported case of nivolumab treatment for chemo-refractory relapsed HL and OC, demonstrating the utility of ctDNA in managing dual malignancies.

## Introduction

The simultaneous occurrence of Hodgkin lymphoma (HL) and gynecologic malignancy, such as ovarian carcinoma (OC), is rare and presents unique challenges in diagnosis, management, and treatment monitoring. Advanced molecular techniques, including the detection of point mutations of candidate genes associated with both lymphoproliferative diseases and solid tumours, enhance diagnostic accuracy and aid in monitoring treatment response.

Circulating tumour DNA (ctDNA), which originates from apoptotic tumour cells and circulates in peripheral blood (PB), offers a minimally invasive and effective tool for detecting residual disease in both malignancies. In such cases, nivolumab—PD-L1 (programmed death ligand 1) monoclonal antibody approved for treating various cancers, including HL and OC—offers a unified therapeutic option. By enhancing T-cell-mediated immunity, nivolumab can effectively target both conditions. However, this immune activation may also result in false-positive findings on PET/CT scans, complicating treatment decisions.

We present the case of a woman diagnosed with concurrent relapsed HL and OC, treated with nivolumab and monitored using both standard PET/CT imaging and PB ctDNA analysis. This case underscores the potential of ctDNA as a method for cancer detection across unrelated malignancies occurring concurrently while highlighting the challenges of PET/CT interpretation during immunotherapy.

## Case summary

**Fig. 1:**
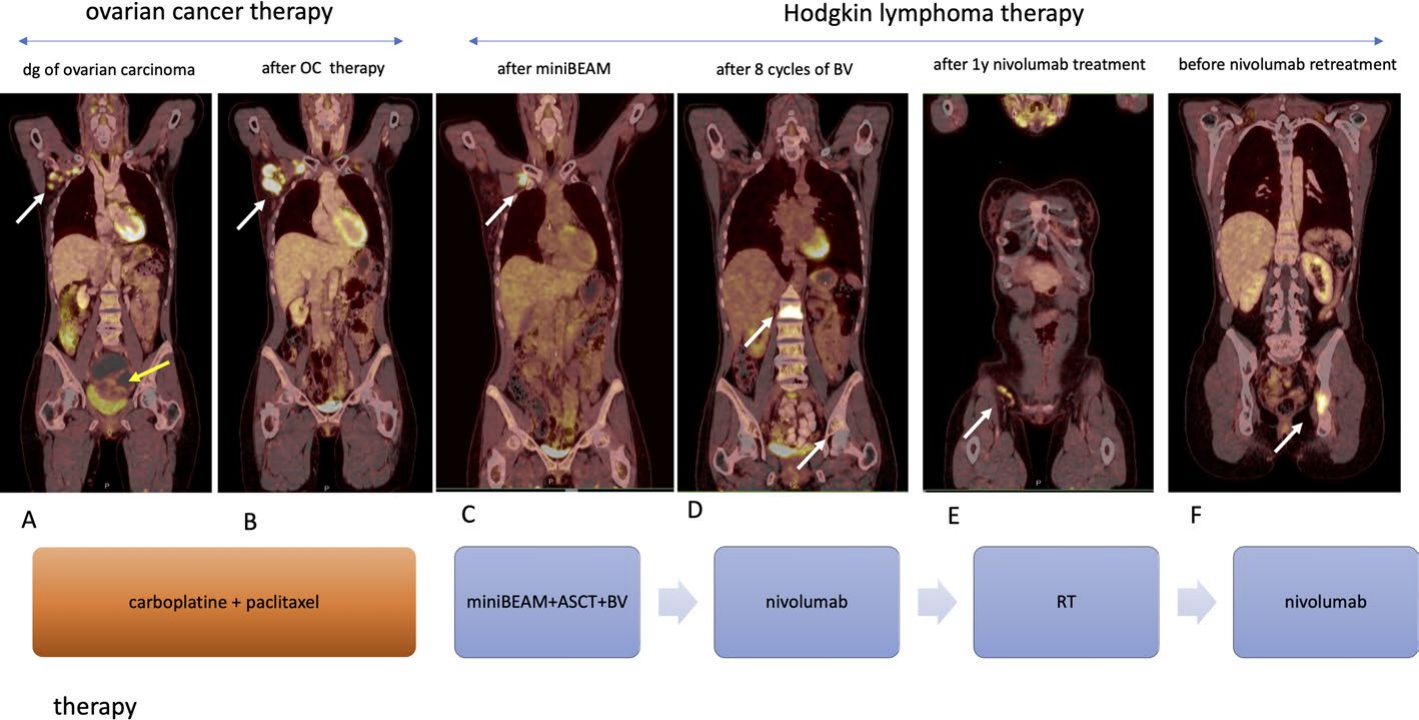
PET/CT findings during the treatment. White arrows—HL, yellow arrow—OC. **A** PET/CT scan at the time of diagnosis of ovarian carcinoma showing metabolically active axillar lymph nodes and heterogenous pelvic mass. **B** PET/CT scan after treatment of OC and before therapy of Hodgkin lymphoma showing complete regression of the pelvic mass and progression of axillar lymphadenopathy. **C** PET/CT scan after 2 cycles of chemotheraphy miniBEAM with ASCT **D** PET/CT scan after eight cycles of brentuximab-vedotin detected high metabolic activity in new locations, showing evident progression of the disease. **E** PET/CT scan after one year of treatment with nivolumab, with minimal residual involvement of inguinal lymph node. **F** PET/CT scan showing hypermetabolic lesion in left hip, before nivolumab retreatment

A 42-year-old woman in remission from intermediate-stage nodular-sclerosis classic Hodgkin lymphoma (cHL) for nearly 7 years was referred to our department in August 2019 with enlarged axillar lymph nodes (LNs). Staging PET/CT scan detected parasternal, mediastinal and right-sided enlargement of LNs and an asymptomatic pelvic tumour mass (Fig. 1A). Patient underwent surgical pelvic tumour debulking combined with hysterectomy and adnexectomy. Histopathologic examination revealed an advanced high grade serous OC (Fédération Internationale de Gynécologie et d’Obstétrique IIIA, pT3aN0M0R0). Concurrent axillary LN biopsy confirmed cHL relapse. The diagnosis of OC has been prioritized and the patient received 4 cycles of adjuvant chemotherapy (paclitaxel in combination with carboplatin) out of 6 planned cycles. Therapy was terminated in January 2020 due to intolerance and complications. Patient achieved complete remission of OC with persistent supradiaphragmatic LN enlargement with high metabolic activity according to follow-up PET/CT scan in July 2020 (Fig. 1B). Rebiopsy of axillary lymph node confirmed diagnosis of cHL, excluding metastasis of OC. Due to the patient’s heavily pretreated disease course and cumulative toxicity from prior therapies, she received two cycles of miniBEAM chemotherapy (carmustine, cytarabine, etoposide, and melphalan) as a less intensive salvage regimen for relapsed cHL. This was followed by autologous stem cell transplantation, after which a partial metabolic response was confirmed by PET/CT in December 2020.

**Fig. 2:**
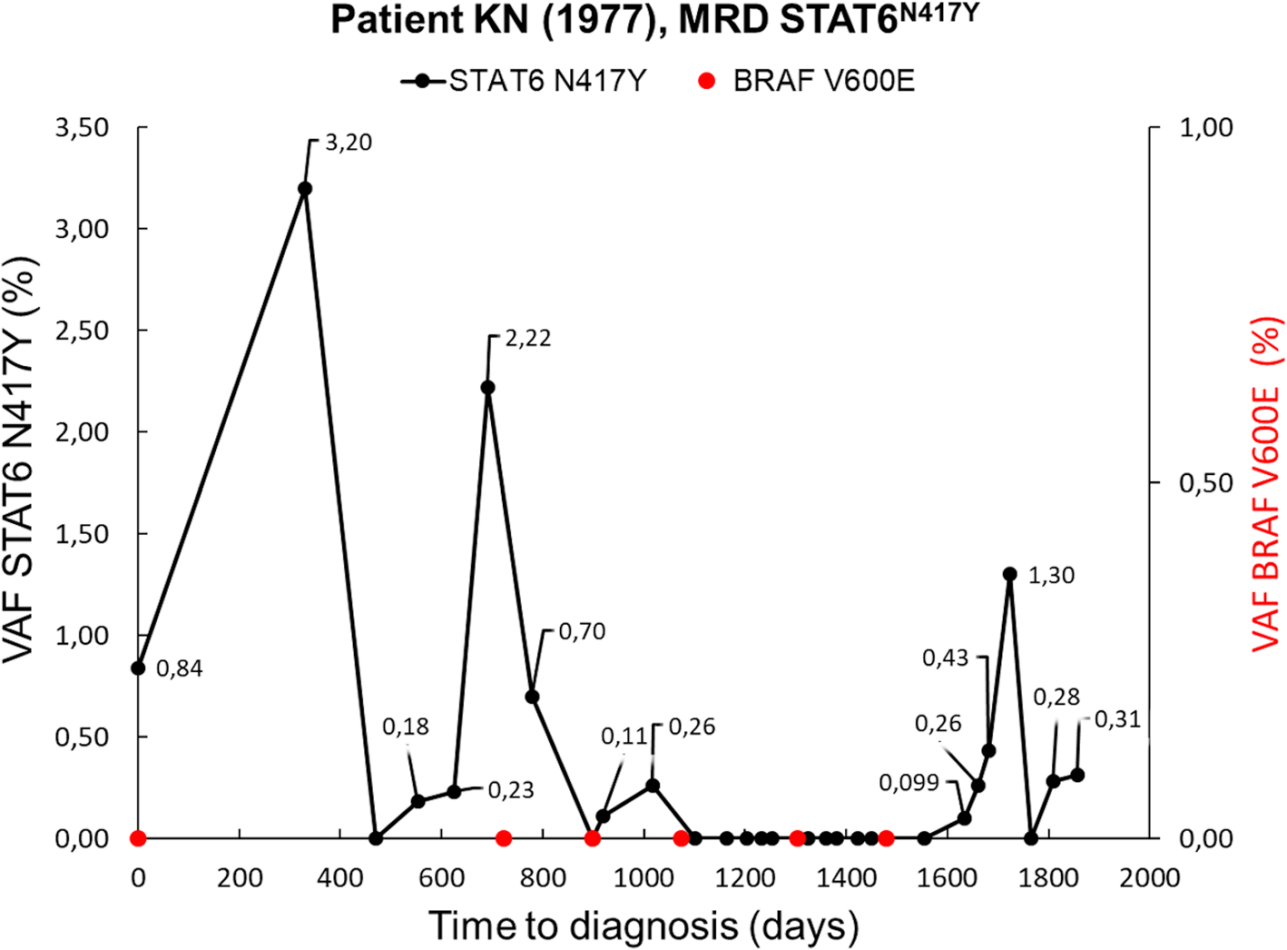
MRD monitoring of both cancers during the anti PD-1 therapy

**Fig. 3:**
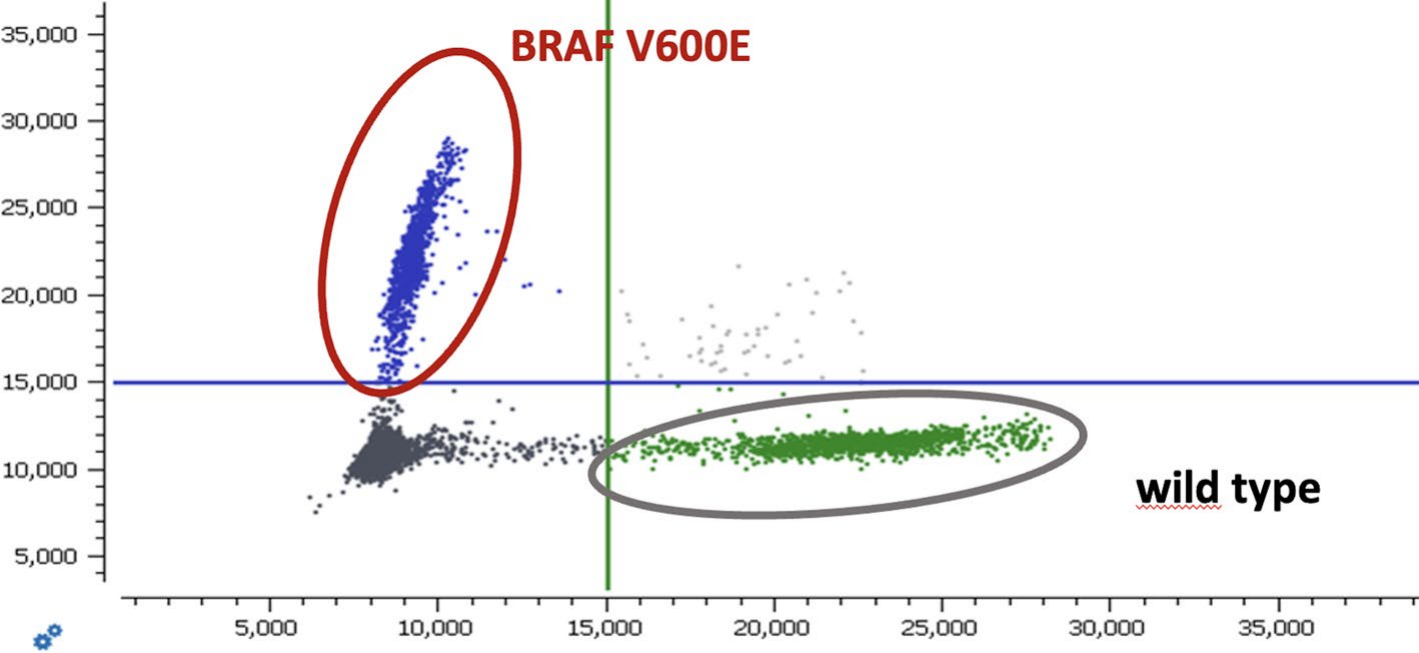
Digital PCR detection of BRAF V600E mutation in analyzed ovarian tumor tissue, VAF 48.9%

Due to high risk of cHL relapse, the patient immediately started maintenance therapy with brentuximab-vedotin (BV), dosed 1,8 mg/kg every three weeks [1]. After eight cycles, PET/CT showed new FDG-avid sites of disease, including axillar, inguinal LNs and bone lesions (Fig. 1C, D). In August 2021 treatment with PD-L1 inhibitor nivolumab (flat dose 240 mg) was initiated. In the present case, nivolumab was initiated specifically for relapsed Hodgkin lymphoma. Restaging PET/CT scan after 12 doses of nivolumab showed tumour regression in all locations. Next scan was performed after one year of treatment (Fig. 1E), with minimal residual involvement of inguinal LN, low dose involved-site radiotherapy 30 Gy of the residual LNs was indicated for possible abscopal effect in October 2022. Following PET/CT scan in March 2023 detected regression in all localities except the axillary nodes. Nivolumab (36th dose) was terminated on August 2023 and patient was indicated to involved-field radiotherapy 36 Gy of the PET positive small axillary LNs. Given the high risk of the OC relapse and difficult assessment of the response during immune checkpoint inhibitor (ICI) therapy we have collected blood samples for the analysis of circulating tumour DNA (ctDNA) to monitor both cancers (Fig. 2). For ovarian carcinoma, presence of the BRAF V600E mutation as the marker was detected from archival paraffin-embedded ovarian tissue collected at the time of diagnosis using next generation sequencing (NGS) as a standard method for sequencing of DNA or RNA, while excluding other mutations, including BRCA 1/2 (Fig. 3). Similarly, we utilized NGS to analyze ctDNA from cryopreserved plasma aliquots focusing on detecting mutations specific to cHL. CtDNA was extracted using the QiaAmp Circulating nucleic acid kit and specific NGS panel covering coding sequences of 13 selected genes (B2M, CD36, CIITA, GNA13, HIST1H1E, ITPKB, NFKBIE, PTPN1, SOCS1, SPEN, STAT6, TNFAIP3, XPO1) was designed. Sequencing was performed on a NovaSeq6000 (Illumina), for desired coverage up to 5000x. Data were analysed with the SureCall software (Agilent Technologies, USA) with sensitivity of 0.5% variant allele frequency (VAF). Variants above 50% were excluded from the analysis as potentially non-tumour origin. Our analyses identify a Hodgkin-driven compound mutation in the STAT6 gene N417Y/N421S. Recurrent mutations in the DNA-binding domain of STAT6 have previously been reported as one of the most frequent in classical Hodgkin lymphoma.

These two mutations were used for MRD monitoring of both cancers during the anti-PD-L1 therapy. In addition, we utilized the digital PCR technique (dPCR) for ctDNA quantification, as a method which is a less time consuming and requires smaller concentration of ctDNA in comparison with NGS. For dPCR, we used a QIAcuity Digital PCR System with a detection threshold of 0.1% VAF for positivity sample as it was described before [2]. 

Three months after involved-field radiotherapy of the PET positive small axillary LNs, peripheral blood ctDNA dPCR assay detected borderline positive signal for STAT6 gene N417Y/N421S which increased in 4 weeks follow-up. Concurrently, the follow up PET/CT revealed hypermetabolic lesions in the sternum and acetabulum regions outside the irradiated field consistent with lymphoma progression (Fig. 1F). CtDNA for BRAF gene remained undetectable. For clinically confirmed relapsed cHL soon after, nivolumab was re-started. Regarding the OC, patient has been in 5 years ongoing remission with undetectable CA125 and no subdiaphragmatic findings on surveillance PET/CT.

## Discussion

The coincidence of the relapsed HL and a secondary cancer possess a great challenge for the treating physician. Ovarian carcinomas are a heterogenous group of neoplasms that are subclassified by the degree of the differentiation. Each major histological type has characteristic genetic defects that deregulate specific signaling pathways in the tumour cells [3]. Mutations in BRAF, mitogen-activated protein kinase (MAPK) cascade component, are common in serous ovarian tumours [4]. BRAFV600E is a point mutation in the BRAF gene. There is a high incidence of BRAF mutation in OC, especially in low grade serous tumours and the presence of BRAF mutation carries favourable prognosis [5]. Cytoreductive surgery and platinum- or taxane-based chemotherapies serve as a standard curative intent treatment of OC, yet ~ 70% of patients with advanced OC who achieve remission ultimately experience relapse. There are only few effective treatments for these patients [6]. ICI including the anti-PD1 antibody nivolumab demonstrated some clinical efficacy and tolerability in patients with platinum-resistant ovarian cancer, but robust data showing benefits in overal survival are still lacking [7]. 

Hodgkin lymphoma, a germinal center B-cell lymphoma, consists of typical Hodgkin and Reed Sternberg cells (HRS cells) characterized with high expression of the ligands shaping the HL immunosupresive microenvironment like PD-L1 and PD-L2 [8]. Somatic mutations and/or amplification of genes involved in specific signaling pathways (NF-κB, PI3K/AKT, NOTCH, and JAK/STAT) are important in pathogenesis of Hodgkin lymphoma [9–11]. Majority of the patients reach complete remission with multi-agent chemotherapy, however, in 20–30% patients with HL relapses occur. In the context of relapsed cHL, an important consideration is the risk of subsequent consecutive relapses [12–14]. PET/CT scan is the main tool for detecting early response and treatment tailoring. However, the functional imaging has reached its limit in terms of depth of CR, and on the other hand might provide false-positive results due to the tumor flare in patients treated with T-cell activating therapies. Assessing the response in blood compartment beyond the sensitivity of interim PET/CT imaging using molecular methods could be important improvement in HL management [15, 16]. 

Cell free DNA (cfDNA) refers to fragments of DNA that shed from cells and can be detected circulating in the peripheral blood as genetic material. CfDNA can originate from both normal or abnormal cells [17]. Cell free DNA released from tumour cell termed ctDNA is detectable from peripheral blood, as “liquid biopsy”. Main advantages of liquid biopsy are non-invasivity and easy access for longitudinal monitoring. Liquid biopsy could detect genetic mutations from ctDNA and could be useful for diagnostics, evaluating treatment response or detect MRD and early relapse. Tumours are often heterogenous, consisting of variety of tumour clones with different mutations, that can lead to challenging diagnostics or resistance to therapies. In HL, ctDNA shedding is abundant despite relative rarity of RS cells due to tumour activity of DNA shedding enzyme ABC [18]. Among many cHL ctDNA mutations, STAT6 was identified as the most frequently mutated gene [19]. 

We discuss the co-occurrence of two clonally unrelated malignancies in a single patient. Specifically, we identified a BRAF V600E mutation in the ovarian tissue collected at the time of OC diagnosis. However, this mutation was no longer detectable in ctDNA at the time of anti-PD1 therapy, likely due to ongoing remission of OC. Utilizing digital PCR, we detected only wild-type form of BRAF in ctDNA. Additionally, further analysis of ctDNA with NGS assay detected the mutation specific for HL in STAT6 gene (N417Y/N421S). Furthermore, ctDNA peripheral blood surveillance during follow ups by dPCR to detect STAT6 gene mutation highlighted the important capacity of this assay to detect early relapse, which otherwise was not clinically apparent.

In conclusion, we have presented to our knowledge the first described case being treated with immune check-point inhibitor therapy for chemo-refractory relapse of cHL and concomitant OC occurring as a secondary cancer. This case report exemplifies the unique advantages and utility of ctDNA detection and monitoring to aid complex clinical decision making and therapy evaluation of concurrent malignancies.

## Data Availability

The dataset analyzed in this study is available from the corresponding author upon reasonable request. The corresponding author had full access to all data in the study and takes responsibility for the integrity of the data and the accuracy of the data analysis.
